# Prevalence of Four Sarcopenia Criteria in Older Patients with Cancer, and Their Predictive Value for 6-Month Mortality: The NutriAgeCancer National Prospective Cohort Study

**DOI:** 10.3390/nu15061508

**Published:** 2023-03-21

**Authors:** Claudia Martinez-Tapia, Kevin Rougette, Virginie Fossey-Diaz, Tristan Cudennec, Cherifa Taleb, Laurent Balardy, Cécile Mertens, Nathalie Mitha, Michael Bringuier, Karin Maley, Sandrine Estivin, Valérie Quipourt, Florence Canoui-Poitrine, Capucine Baldini, Johanne Poisson, Elena Paillaud

**Affiliations:** 1 Univ Paris Est Créteil, INSERM, IMRB, F-94010 Créteil, France; 2 Assistance Publique-Hôpitaux de Paris (AP-HP), Paris Cancer Institute CARPEM, Georges Pompidou European Hospital, Department of Geriatric Medicine, Oncogeriatric Coordination Unit (UCOG), F-75015 Paris, France; 3 AP-HP, Hôpital Bretonneau, Service de Gériatrie, UCOG Paris Nord, F-75018 Paris, France; 4 AP-HP, Hôpital Ambroise-Paré, Department of Geriatrics, UCOG Paris Ouest, F-92100 Boulogne-Billancourt, France; 5 AP-HP, Hôpital René-Muret, Service de Gériatrie, HUPSSD, UCOG Paris Seine Saint Denis, F-93270 Sevran, France; 6 Centre Hospitalier Universitaire de Toulouse, Geriatric Department, Internal Medicine and Oncogeriatry Unit, UCOG Midi-Pyrénées, F-31000 Toulouse, France; 7 CHU Bordeaux, Clinical Gerontology Department, Aquitaine Interregional UCOG, F-33000 Bordeaux, France; 8 CHU de Grenoble Alpes, Geriatric Medicine Department, UCOG Arc Alpin, F-38700 Grenoble, France; 9 Institut Curie, PSL Research University, Department of Medical Oncology and Department of Supportive Care, UCOG Paris Ouest, F-92210 Saint-Cloud, France; 10 Groupe Hospitalier Diaconesses-Croix-Saint-Simon, Geriatric Medicine Department, UCOG Paris Est, F-75020 Paris, France; 11 Brest University Hospital, Internal Medicine and Geriatrics, UCOG de Bretagne, F-29200 Brest, France; 12 Dijon University Hospital, Department of Geriatrics and Internal Medicine, Bourgogne Interregional UCOG, F-21000 Dijon, France; 13 AP-HP, Henri-Mondor Hospital, Public Health Department, F-94010 Creteil, France; 14 Drug Development Department DITEP, Gustave Roussy, F-94800 Villejuif, France; 15 Université Paris-Cité, Inserm, Centre de Recherche sur L’inflammation, UMR 1149, F-75018 Paris, France; 16 AP-HP, Oncogeriatric Coordination Unit (UCOG), Department of Geriatric Medicine, Georges Pompidou European Hospital, Paris Cancer Institute CARPEM, F-75015 Paris, France

**Keywords:** sarcopenia, assessment criteria, older adult, cancer, mortality

## Abstract

Older cancer patients have an elevated risk of sarcopenia. The aim was to estimate the prevalence of four criteria for sarcopenia case finding, assessment, diagnosis, and severity determination: abnormal strength, assistance with walking, rising from a chair, climbing stairs, and falls (SARC-F), low hand-grip strength (HGS), low arm circumference (AC, a muscle mass proxy), and low physical performance (PP). Sarcopenia (low HGS and AC) and severe sarcopenia (low HGS, AC, and PP) and their predictive values for 6-month mortality were estimated in the whole population and by metastatic status. We analyzed data from the NutriAgeCancer French nationwide study of cancer patients aged ≥70 referred for geriatric assessment before anti-cancer treatment. We performed Cox proportional hazards analysis for each criterion separately and all criteria combined. Overall, 781 patients from 41 geriatric oncology clinics were included (mean age: 83.1; females: 53%; main cancer types: digestive (29%) and breast (17%); metastases: 42%). The prevalence of abnormal SARC-F, low HGS, a low AC, low PP, sarcopenia, and severe sarcopenia were, respectively, 35.5%, 44.6%, 44.7%, 35.2%, 24.5%, and 11.7%. An abnormal SARC-F and/or low HGS, sarcopenia, and severe sarcopenia were associated with 6-month mortality in patients with metastases (adjusted hazard ratios [95% confidence interval]: 2.72 [1.34–5.49], 3.16 [1.48–6.75] and 6.41 [2.5–16.5], respectively). Sarcopenia was strongly predictive of 6-month mortality in patients with metastatic cancer.

## 1. Introduction

Sarcopenia is a disorder characterized by progressive, generalized skeletal muscle loss, which is associated with several adverse outcomes [1]. Older patients with cancer are exposed to a greater risk of sarcopenia. In addition to physiological changes in muscle mass and function associated with advanced age, older cancer patients are affected by the physical and metabolic effects of the cancer disease itself and its treatment on the skeletal muscle mass [2,3].

The prevalence of sarcopenia in patients with cancer varies significantly according to the type of cancer and stage, the criterion or criteria applied, and the assessment methods used [4]. In a recent systematic review and meta-analysis of eight studies encompassing a total of 5744 patients with breast cancer, the prevalence of sarcopenia was 37.6% overall, 36.3% among patients with non-metastatic cancer, and 55.1% among those with metastatic cancer [5]. In another systematic review of older patients with cancer receiving radiotherapy, the prevalence of sarcopenia ranged from 42.8% to 72% [6].

The identification and assessment of sarcopenia is of particular importance in patients with cancer as it has been significantly associated with a poorer prognosis, an elevated risk of postoperative complications, a longer stay in hospital, and greater chemotherapy toxicity in a wide variety of cancer types [7,8,9,10,11,12].

The European Working Group on Sarcopenia in Older People 2019 (EWGSOP2) recently suggested an operational definition of sarcopenia based on a stepwise clinical path algorithm for case finding, assessment, diagnosis and severity determination [1]. EWGSOP2 advises using the SARC-F questionnaire to screen for individuals at risk of sarcopenia [13]. Probable sarcopenia is then identified by low muscle strength, using hand-grip strength (HGS) or chair stand measurements. Sarcopenia diagnosis is confirmed when low muscle strength and low muscle quantity or quality are present. The EWGSOP2 recommends muscle evaluation by dual-energy X-ray absorptiometry and bioelectrical impedance assessment in routine clinical care. Lastly, indices of physical performance (PP, mainly the Short Physical Performance Battery, the timed-up-and go (TUG) test, and the 400 m walk test) are recommended for assessing the severity of sarcopenia. If the recommended muscle mass measurement methods are not available, the EWGSOP2 suggests using calf circumference as a proxy diagnostic marker in older adults. On the same lines, arm circumference (AC) has been suggested as an alternative index of muscle mass for the identification of sarcopenia [14,15,16].

The associations between mortality and each EWGSOP2 sarcopenia criterion (case finding with SARC-F, muscle strength, muscle mass/quality, and PP) have been studied in several settings, including older patients in general and older patients with cancer [17,18,19]. However, to the best of our knowledge, it is not known whether the combination of an anthropometric muscle mass evaluation method with low HGS is able to predict mortality. Moreover, the use of the EWGSOP2 algorithm (including the SARC-F) to categorize sarcopenia has not previously been studied.

The present study aimed to (i) assess the prevalence of (and relationships between) the four EWGSOP2 criteria (corresponding to an abnormal SARC-F result, low HGS, a low AC, and an abnormal TUG completion time) in older patients with cancer, (ii) estimate the prevalence of sarcopenia and severe sarcopenia, using an anthropometric proxy measure of muscle mass, and (iii) assess the respective abilities of SARC-F, HGS, AC, sarcopenia, and severe sarcopenia to predict 6-month mortality (overall and by metastatic status), in the population as a whole and in the subpopulations defined by the EWGSOP2 algorithm.

## 2. Materials and Methods

### 2.1. Study Design and Patients

We analyzed data on patients aged 70 or over from the NutriAgeCancer study, which is a French survey of a nationwide network of geriatric oncology clinics. All the patients had been referred to a geriatric oncology clinic for a geriatric assessment (GA) before anti-cancer treatment [20]. Patients who were unable to understand information about the study and/or give their consent to participation were not included in the study.

In a cross-sectional analysis, we included patients with available data on the four criteria. For the survival analysis, we included patients for whom follow-up data were also available.

All participants gave their informed consent before inclusion in the study. The study was approved by the local independent ethics committee (CCP Ile de France XI, Paris, France; reference: IDRCB 2017-A01397-46, 17035) and is registered at ClinicalTrials.gov (NCT03390816).

### 2.2. Data Collection

Demographic data (age, sex, and outpatient status), data related to cancer (site, metastatic status, and cancer treatment decision: curative, palliative or supportive care alone), and the Eastern Cooperative Oncology Group Performance Status were registered at baseline [20]. Geriatric information in different health domains was evaluated: functional status (activities of daily living [21]), mobility (TUG test completion time [22]), cognitive status (the Mini Mental State Examination score (MMSE) [23] and the presence or absence of a physician-diagnosed cognitive disorder), mood (mini-Geriatric Depression Scale score [24]), comorbidities (the updated Charlson Comorbidity Index [25]), polypharmacy, and nutritional status (i.e., weight loss over the previous 6 months, and the body mass index (BMI)). The risk of frailty was defined as a G-8 score of 14 or less out of 17 [26].

### 2.3. Criteria for Sarcopenia Case-Finding, Assessment, Diagnosis and Severity Determination

#### 2.3.1. The SARC-F Questionnaire

The SARC-F is a five-item self-reported questionnaire that captures the patient’s perception of limitations in strength, walking ability, rising from a chair, stair climbing, and experiences with falls. The score ranges from 0 (best) to 10 (worst), and a total score of 4 or more is considered to be abnormal [13,27].

#### 2.3.2. Hand-Grip Strength

Muscle strength was assessed through the patient’s HGS, as measured twice for the dominant hand using a Jamar^®^ (Model J00105; Sammons Preston, Bolingbrook, IL, USA) hand-held hydraulic dynamometer [28]. Low HGS was defined as a value of <27 kg for men and <16 kg for women [1].

#### 2.3.3. Arm Circumference

Arm circumference was used as a proxy marker of muscle mass. It was measured using a flexible, non-stretch tape laid at the midpoint between the acromion and olecranon processes of the upper arm. Cut-off points varies according to different studies; mostly 27 cm and 29 cm are used, according to optimal discrimination or lower tertile or quartile. In our study, the lower tertile was ≤ 24 cm, which was much lower than other cut-off points used in the literature. Low AC was defined as a value of <26 cm for men and <25 cm for women, according to the sex-specific median values in this population.

#### 2.3.4. The TUG Test

The patient was asked to stand up from a chair, walk 3 m, and return to sit down [22]. Low PP was defined as an abnormal TUG completion time of ≥20 s [1] or the inability to perform the test.

Probable sarcopenia was defined as a low HGS [1]; sarcopenia was defined as low HGS and a low AC; and severe sarcopenia was defined as low HGS, a low AC, and low PP.

### 2.4. Outcome

The outcome was mortality 6 months after geriatric assessment, which was determined by telephoning the patients or family members or obtained from medical records.

### 2.5. Statistical Analysis

We analyzed demographic and clinical characteristics and the results of the GA. Quantitative variables were expressed as the median (interquartile range) and categorical variables were expressed as the frequency (percentage). The prevalence and the corresponding 95% confidence interval (CI) were estimated for an abnormal SARC-F, low HGS, a low AC, and low PP. The overlaps between the four criteria were evaluated. Their associations were assessed using the chi-square test.

In a survival analysis, the cumulative survival probability for each criterion group was calculated using the Kaplan–Meier method and compared using the log-rank test. Multivariate Cox proportional hazard analyses were performed for each criterion after adjustment for age (≥85), cancer site, metastatic status, cancer treatment decision (curative, palliative, or supportive care), low body weight (BMI <22 kg/m^2^), weight loss in the previous 6 months (>5%), the number of prescription medications per day, outpatient status, and cognitive impairment (an impaired MMSE score (<24) or a physician-diagnosed cognitive disorder) [20] overall and by metastatic status. On the basis of these results and the literature data [1], we then created two composite variables. The first classified each patient with regard to sarcopenia, according to the EWGSOP2 consensus definition: (i) no sarcopenia (normal HGS), (ii) probable sarcopenia (low HGS only), (iii) sarcopenia, or (iv) severe sarcopenia. The second composite variable included the SARC-F result in the definition: (i) a normal SARC-F score (<4) and normal HGS (the reference category), (ii) an abnormal SARC-F score and/or low HGS, (iii) sarcopenia, and (iv) severe sarcopenia. Interaction terms were assessed using the Wald test. Multivariate Cox proportional hazard models were created for each composite variable, overall and by metastatic status. Hazard ratios (HRs) and their 95% CIs were computed. Discrimination was assessed using Harrell’s C-index [29] and the K-concordance statistic [30]. C-index values of 0.70–0.79 and 0.80–0.89 correspond to good and very good discriminative ability, respectively [31]. A higher K-concordance statistic indicates better discriminative ability. Calibration was assessed using the slope test: a *p*-value above 0.05 indicated good calibration. The proportional hazard assumption was tested by Schoenfeld residual plots and was met.

In a sensitivity analysis addressing one of the study’s objectives, we used multivariate Cox proportional hazard models to analyze the EWGSOP2′s algorithm in the different subpopulations as a function of the SARC-F and HGS results.

All tests were two-tailed, and the threshold for statistical significance was set to *p* < 0.05. Analyses were performed using the Stata software (version 16, StataCorp. 2019. Stata Statistical Software: Release 16. StataCorp LLC., College Station, TX, USA).

## 3. Results

### 3.1. Characteristics of the Study Population

In total, 781 patients were included in the cross-sectional analysis and 640 were included in the survival analysis (Supplementary Figure S1). Mean age was 83.1 ± 5.99, 53% were women, and 42% had metastases (Table 1). The most frequent cancers were digestive tract cancers (29%) and breast cancer (17%). Curative treatment was decided for half the patients. A high proportion of patients (87%) were at risk of frailty (G-8 score ≤14). Almost half of the patients (47%) had lost more than 5% of their body weight in the last 6 months. Cognitive impairment and a risk of depression were present in around 40% of the patients.

### 3.2. Prevalence of and Relationships between Criteria for Sarcopenia

The prevalence of an abnormal SARC-F result, low HGS, a low AC and low PP were respectively 35.5% [95%CI: 32.1–38.9%], 44.6% [41.0–48.1%], 44.7% [41.2–48.3%], and 35.2% [31.9–38.7%] (Table 1).

We found strong associations between SARC-F, HGS and the TUG test result (*p* < 0.0001; Table 2). AC was strongly associated with HGS (*p* < 0.0001), less strongly associated with the SARC-F (*p* = 0.047) and not associated with the TUG test result (*p* = 0.33).

**Figure 1:**
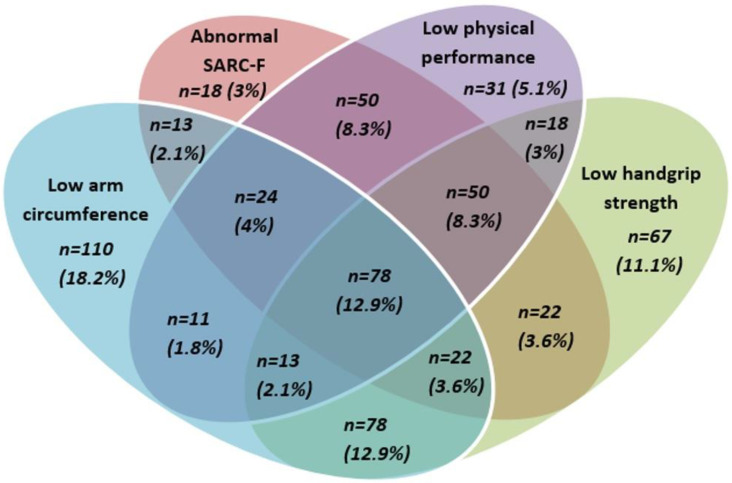
Overlap between four criteria for probable sarcopenia in older patients with cancer (*n* = 605).

Of the 781 patients in the study, 176 (22.5%) had normal scores for the four criteria and 605 (77.5%) had at least one abnormal criterion: 226 (28.9%) had only one abnormal criterion, 192 (24.6%) had two abnormal criteria, 109 (14%) had three abnormal criteria, and 78 (10%) had four abnormal criteria (Figure 1).

Sarcopenia was present in 191 patients (24.5%; 95%CI: 21.5–27.6%). Almost half of these (*n* = 91; 47.6%) presented severe sarcopenia (11.7% of the whole population; 95%CI: 9.5–14.1%). The prevalence of sarcopenia was similar in patients with metastatic cancer and those with non-metastatic cancer (24.7% vs. 24.4%, respectively).

### 3.3. The Survival Analysis

The median follow-up time was 6 months (interquartile range: 5.07–6.97). The 6-month overall mortality rates [95%CI] for the whole population with follow-up data (*n* = 640), for patients with non-metastatic cancer (*n* = 364), and for patients with metastatic cancer (*n* = 276) were, respectively, 23.8% [20.4–27.5%], 15.2% [11.7–19.7%], and 34.7% [29.0–41.1%].

**Figure 2:**
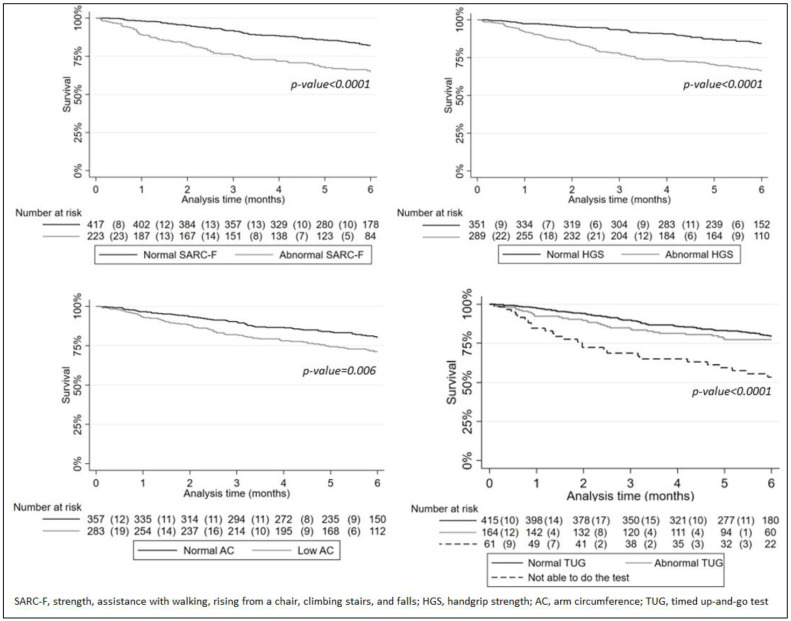
Kaplan–Meier survival curves for four criteria for probable sarcopenia.

Kaplan–Meier curves for each criterion showed significantly lower 6-month survival rates for patients with abnormal or low scores (*p* < 0.006; Figure 2).

In a multivariate analysis, the SARC-F, HGS, and TUG test result were independently associated with 6-month mortality in the study population as a whole, while AC was not significant (adjusted HR (aHR) = 1.49; 95%CI: 0.96–2.32; *p* = 0.074). The 6-month mortality risk for patients with an abnormal SARC-F was similar to that for patients with a low HGS when compared with patients with a normal SARC-F or a normal HGS (Table 3); the aHRs were 1.82 [95%CI: 1.20–2.77] for an abnormal SARC-F and 1.79 [1.16–2.75] for a low HGS. Concerning the TUG test result, only patients unable to perform the test had a higher risk of death than patients with a normal TUG test result (aHR = 2.29 [1.32–3.97]). With regard to metastatic status, statistically significant associations with 6-month mortality were observed in patients with metastatic cancer but never in patients with non-metastatic cancer.

When considering the first composite variable (the EWGSOP2 consensus definition of sarcopenia), only patients with sarcopenia or severe sarcopenia had a higher 6-month mortality risk than patients without sarcopenia (Table 4). When analyzing the composite variable that included the SARC-F in the definition of sarcopenia, patients with an abnormal SARC-F and/or low HGS, those with sarcopenia, and those with severe sarcopenia had a higher 6-month mortality risk than patients with normal SARC-F and HGS results (Table 4); we observed a significant linear trend for the HRs (*p* < 0.001). Both models showed very good discriminative ability (Harrell’s C-index >0.8 and K-concordance statistic ≥0.78) and good calibration (*p* > 0.5).

**Figure 3:**
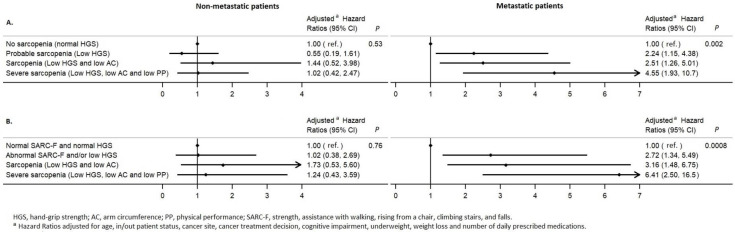
Multivariate Cox analyses of the association between two composite variables for sarcopenia and 6-month mortality, by metastatic status: (**A**) A composite variable corresponding to the EWGSOP2 definition of sarcopenia; (**B**) A composite variable that included the SARC-F in the definition.

After stratification by metastatic status, we did not observe associations in patients with non-metastatic cancer but found associations similar to those described above in patients with metastases. The aHRs were higher—particularly in patients with severe sarcopenia (Figure 3).

The C-index and the K-concordance statistic were, respectively, 0.78 and 0.75 for the model with the EWGSOP2 composite variable, and 0.79 and 0.76 for the model including the SARC-F. Both models had a greater discriminative ability than the baseline model (C-index: 0.76; K-concordance statistic: 0.73). The results did not change greatly after the exclusion of patients who were not able to perform the TUG test (*n* = 61) (Supplementary Figure S2).

### 3.4. Sensitivity Analysis

Of the 640 patients with available follow-up data, 223 had an abnormal SARC-F score, and 136 of the 223 had low HGS (i.e., probable sarcopenia). In the multivariate Cox model of the population of 223 patients with an abnormal SARC-F, HGS was not predictive of 6-month mortality (Supplementary Table S1). Overall, 76 of the 136 patients with probable sarcopenia had a low AC. In the multivariate Cox model, a low AC was predictive of 6-month mortality (aHR = 2.81 [95%CI: 1.3–6.2]; *p*-value = 0.01). The effect was stronger in the metastatic group (Supplementary Table S1). The model’s C-index was 0.77 with AC and 0.71 without.

## 4. Discussion

The objectives of the present study of older patients with cancer were to (i) assess the prevalence of four EWGSOP2 criteria for the assessment of sarcopenia (namely an abnormal SARC-F score, a low HGS, a low AC and a low PP), (ii) evaluate the relationships between these criteria and (iii) assess each criterion’s ability to predict 6-month mortality. The respective prevalences were similar and ranged from 35.2% (low PP) to 44.7% (a low AC). Most of the associations between the criteria were strong. More than 75% of the population presented at least one abnormal criterion. One-quarter of the patients presented sarcopenia (severe, in almost half of these cases). An abnormal SARC-F score, low HGS, and inability to perform the TUG test were independently associated with poor survival. When the criteria were combined in two composite variables (one based on the EWGSOP2 consensus definition and the other including the SARC-F in the categorization), our results showed a graded relationship between worsening categories and a higher mortality rate in the study population as a whole and in patients with metastatic cancer.

In the present study, the prevalence of probable sarcopenia was 44.6% (according to the EWGSOP2 definition, with low HGS) or 58% (according to the presence of an abnormal SARC-F and/or low HGS). Only one other study (a study of patients with cancer having undergone major surgery; mean ± standard deviation age: 58.7 ± 14.0, i.e., younger than our patients) combined both criteria in the definition of probable sarcopenia (i.e., the presence of both an abnormal SARC-F result and low HGS) and found a prevalence of 6.3% among the 111 older study participants (whose age was not defined) [32]. This prevalence is much lower than ours—probably because Behne et al. (i) combined the two criteria and (ii) studied relatively young candidates for major oncological operations and who were therefore unlikely to have advanced disease and sarcopenia.

In our study, the overall prevalence of sarcopenia was 24.5%. Some researchers have investigated the prevalence of sarcopenia in older adults or in patients with cancer (mostly in patients undergoing surgical procedures [33,34,35]), but few have focused on older patients with cancer. In a study that included 108 patients with cancer aged ≥60 (mean ± standard deviation age: 70.6 ± 7.4; females: 52.3%, main cancer types: colorectal (27.8%) and gastric (22.2%); advanced cancer: 54.6%), the prevalence of sarcopenia was 24.1% according to the EWGSOP2 definition and 25.9% when using the calf circumference as a proxy for muscle mass [36]. These prevalence values are very similar to those found in our study when using the AC as a proxy marker of muscle mass. Furthermore, AC has been shown to have very good discriminative ability for muscle mass or sarcopenia [14,15,16]. In a study of community-dwelling middle-aged and older adults, AC was strongly correlated (r ≥0.7) with an appendicular skeletal muscle mass index and had very good discriminative ability (vs. the EWGSOP2 as the reference standard) [14]. The sensitivity and specificity were, respectively, 87.9% and 71.2% in men and 82.4% and 74.1% in women [14]. In a study of 411 community-dwelling adults aged 60 or over, AC was the best predictor (relative to the EWGSOP2 definition, and ahead of calf circumference) of the presence of sarcopenia (sensitivity: 100%; specificity: 77.34%) [15].

The four criteria used in the assessment of sarcopenia have been investigated with regard to various health outcomes. The SARC-F score has been shown to predict mortality in a variety of populations [17,32,37,38]. In a study of 256 older (age ≥60) adults with various cancer types, an abnormal SARC-F score was independently associated with worse overall survival (aHR = 2.98; 95 CI: 1.1–8.3; *p* = 0.04) [17]. In a study of 220 patients with cancer undergoing major surgical procedures (mean age, 58.7 ± 14 years; including 111 (50.5%) older patients), those with an abnormal SARC-F score and low HGS had a higher risk of death (aHR = 5.8; 1.5–22.6; *p* = 0.011) [32]. Indeed, patients with low muscle strength are at greater risk of adverse health outcomes and poor PP. In a recent systematic review and meta-analysis of 25 studies encompassing 8109 older adults (aged ≥60) with various types of cancer, higher levels of physical function (TUG: HR = 0.40; 95%CI: 0.31–0.53; *p* < 0.001; HGS: HR = 0.61; 95%CI: 0.43–0.85; *p* = 0.004) were associated with a lower risk of all-cause death when compared with lower levels of physical function [18]. Some researchers have investigated a low AC in older adults and found it to be a better predictor than low calf circumference [19] of an elevated mortality risk [39,40]. To the best of our knowledge, the predictive value of a low AC has not been studied in older adults with cancer.

In the present study, we found that two composite variables with increasing risk classes had a graded relationship with 6-month mortality (*p* < 0.0001) in the overall study population and in the subgroup of patients with metastatic cancer. Sarcopenia in cancer is caused by systemic, cytokine-mediated inflammation, which in turn creates a catabolic state with a net loss of skeletal muscle tissue. Consequently, a gradual, generalized loss of skeletal muscle can be observed as the cancer progresses [41]. Partly in contrast to our present results, a systematic review of eight studies encompassing a total of 5744 patients with breast cancer found that sarcopenia also had a negative effect on overall survival in patients with non-metastatic cancer [5]. In our study, the low number of events in the non-metastatic group might have led to a lack of power for the detection of a statistically significant association between sarcopenia and mortality. Longer-term follow-up might be more appropriate in this group of patients.

When applying the EWGSOP2′s algorithm, we did not find that HGS provided additional predictive value for 6-month mortality among patients identified as being at risk of sarcopenia by the SARC-F score. This might be due because the SARC-F captures data on the patient’s strength by asking whether they have difficulty lifting and carrying a 10-pound (4 kg) objects. In the multivariate analyses, the SARC-F and the HGS gave similar HRs in the overall study population. In contrast, a low AC provided additional predictive value for 6-month mortality in patients with probable sarcopenia. A diagnostic confirmation in patients with probable sarcopenia is therefore important for the prognosis.

### 4.1. Strengths and Limitations

NutriAgeCancer is a large, nationwide survey that included patients with various cancer types and stages and collected data on a great variety of geriatric and oncologic variables. To the best of our knowledge, our study is the first to provide a detailed description of the various criteria involved in the EWGSOP2 consensus assessment of sarcopenia in a population of older cancer patients. Furthermore, we evaluated the ability of two composite variables for sarcopenia categorization (one according to the EWGSOP2 and another including the SARC-F) to predict 6-month mortality. Our study also had some limitations. Selection bias was possible because all the study participants had been referred for GA. Lastly, we did not measure muscle mass directly (e.g., with dual-energy X-ray absorptiometry or bioelectrical impedance) and so were unable to compare our proxy model (arm circumference) with direct measurements. Other relevant anthropometric measures for muscle mass such as the calf circumference was not available in our database, either.

### 4.2. Implications for Practice

Despite the growing body of evidence for sarcopenia’s ability to predict death, this variable’s applicability in clinical practice is limited by the complexity of its diagnosis and the measurements of each component criterion. Screening for sarcopenia with alternative (anthropometric) methods might therefore be of value when precise muscle mass measurements are not available. Our present results show that the anthropometric measurement of sarcopenia was strongly predictive of 6-month mortality in older patients with cancer.

Furthermore, although EWGSOP2’s recommendations and results from several studies have increased awareness of sarcopenia and its risks for adverse outcomes, thus promoting early detection, little evidence and recommendations concerning treatment for sarcopenia are available specifically to older patients with cancer. Clinical trials investigating interventions including nutrition and exercise are needed in order to stablish consensual guidelines in this population.

## 5. Conclusions

Each of the four EWGSOP2 consensus criteria for sarcopenia (case finding, assessment, diagnosis, and severity determination) were highly prevalent in our study population of older patients with cancer. Sarcopenia was present in one-quarter of the patients and was a strong predictor of 6-month mortality in patients with metastatic cancer.

## Figures and Tables

**Table 1:** Characteristics of the study population (*n* = 781).

Characteristics		N	%
Age	Mean ± standard deviation	83.1 ± 5.99	
	≥85 years	314	40.2
Sex	Males	365	46.7
Poor performance status	ECOG-PS ≥ 2	334	43.8
Cancer type	Head and neck	34	4.4
	Esophageal/stomach	45	5.8
	Pancreas/liver	62	7.9
	Colorectal	118	15.0
	Prostate	58	7.4
	Urinary tract	67	8.6
	Lung	79	10.1
	Breast	134	17.2
	Gynecological	71	9.1
	Hematological	42	5.4
	Others ^a^	71	9.1
Metastasis		328	42.3
Treatment, missing: *n* = 4	Curative	388	49.9
	Palliative	314	40.4
	Supportive care	76	9.8
Score G-8	Abnormal: ≤14/17	547	86.7
Dependency	ADL ≤5/6	249	32.0
Cognitive impairment	MMSE ≤23/30 or physician-diagnosed disorder	277	38.0
Risk of depression	mini-GDS ≥1/4	284	40.6
Comorbidities	Updated Charlson Comorbidity Index, median (IQR)	5 (3–7)	
Prescription medications	Number taken daily	6 (3–8)	
Malnutrition	MNA score <17/30	105	14.8
Underweight, missing: *n* = 3	BMI <22 kg/m^2^	213	27.3
Weight loss, missing: *n* = 75	>5% in the previous 6 months	335	47.2
Low serum albumin	<35	200	37.9
High serum CRP	>10	242	50.1
SARC-F	Normal: <4/10 pts	504	64.5
	Abnormal: ≥4/10 pts	277	35.5
Hand-grip strength	Median (IQR), men	26 (20–33)	
	Median (IQR), women	18 (12.5–20)	
	Normal: ≥27 kg for men; ≥16 kg for women	433	55.4
	Low: <27 kg for men; <16 kg for women	348	44.6
Arm circumference	Normal: ≥26 cm for men; ≥25 cm for women	432	55.3
	Low: <26 cm for men; <25 cm for women	349	44.7
Timed up-and-go test	Normal: ≤20 sec.	506	64.8
	Abnormal: >20 sec.	199	25.5
	Unable to perform the test	76	9.7

ADL, activities of daily living; BMI, body mass index; CRP, C-reactive protein; ECOG-PS, Eastern Cooperative Oncology Group-Performance Status; GDS, geriatric depression scale; IQR, interquartile range; MMSE, mini mental state examination; SARC-F, strength, assistance with walking, rising from a chair, climbing stairs, and falls. ^a^ Skin (*n* = 31), sarcoma (*n* = 15), unknown origin (*n* = 6), thyroid (*n* = 3), others (*n* = 16).

**Table 2:** Associations between four criteria for probable sarcopenia in older patients with cancer.

		SARC-F			Hand-Grip Strength			Arm Circumference		
		Normal	Abnormal	*p*-Values	Normal	Low ^a^	*p*-Values	Normal	Low ^b^	*p*-Values
		(<4/10 pts)	(≥4/10 pts)							
		N (%)	N (%)		N (%)	N (%)		N (%)	N (%)	
**Hand-grip strength**	Normal	328 (65.1)	105 (37.9)	<0.0001						
	Low ^a^	176 (34.9)	172 (62.1)							
**Arm circumference**	Normal	292 (57.9)	140 (50.5)	0.047	275 (63.5)	157 (45.1)	<0.0001			
	Low ^b^	212 (42.1)	137 (49.5)		158 (36.5)	191 (54.9)				
**Timed** **up-and-go test**	Normal (≤20 s)	431 (85.5)	75 (27.1)	<0.0001	317 (73.2)	189 (54.3)	<0.0001	283 (65.5)	223 (63.9)	0.33
	Abnormal (>20 s)	69 (13.7)	130 (46.9)		99 (22.9)	100 (28.7)		113 (26.2)	86 (24.6)	
	Unable to perform the test	4 (0.8)	72 (26.0)		17 (3.9)	59 (17.0)		36 (8.3)	40 (11.5)	

^a^ <27 kg in men and <16 kg in women; ^b^ <26 cm for men and <25 cm for women.

**Table 3:** Multivariate analysis of criteria for probable sarcopenia as predictors of 6-month mortality.

		Overall Population *N* = 536		Non-Metastatic Cancer *n* = 309		Metastatic Cancer *n* = 227	
Criteria		aHR ^a^	*p*-value	aHR ^a^	*p*-value	aHR ^a^	*p*-value
SARC-F	Normal (score < 4)	1(ref)	0.005	1(ref)		1(ref)	
	Abnormal (score ≥ 4)	1.82 (1.20–2.77)		1.61 (0.78–3.35)	0.20	1.94 (1.10–3.43)	0.022
Hand-grip strength	Normal	1(ref)	0.008	1(ref)		1(ref)	
	Low (<27, men; <16, women)	1.79 (1.16–2.75)		0.90 (0.44–1.85)	0.78	2.61 (1.52–4.47)	<0.0001
Arm circumference	Normal	1(ref)	0.074	1(ref)		1(ref)	
	Low (<26, men; <25, women)	1.49 (0.96–2.32)		1.04 (0.49–2.17)	0.92	1.73 (0.98–3.04)	0.057
Timed up-and-go test	Normal (≤20 s)	1(ref)	0.004	1(ref)	0.06	1(ref)	0.13
	Abnormal (>20 s)	0.90 (0.55–1.48)	0.67	0.49 (0.19–1.23)	0.13	1.04 (0.56–1.94)	0.90
	Unable to perform the test	2.29 (1.32–3.97)	0.003	1.51 (0.63–3.61)	0.36	2.32 (1.01–5.32)	0.048

SARC-F, strength, assistance with walking, rising from a chair, climbing stairs, and falls. ^a^ Hazard ratios adjusted for age, in/out patient status, cancer site, metastatic status, cancer treatment, cognitive impairment, underweight, weight loss, and number of prescription medications per day.

**Table 4:** Adjusted hazard ratios for two composite variables for sarcopenia and 6-month mortality.

Composite Variables for Sarcopenia	aHR ^a^ [95%CI]	*p*-Value
**According to the EWGSOP2 definition**		
No sarcopenia (normal HGS)	1.00 (ref)	0.009
Probable sarcopenia (Low HGS)	1.31 [0.75–2.30]	
Sarcopenia (Low HGS and low AC)	2.07 [1.19–3.61]	
Severe sarcopenia (Low HGS, low AC, and low PP)	2.52 [1.38–4.62]	
**Including the SARC-F in the definition**		
Normal SARC-F and normal HGS	1.00 (ref)	0.003
Abnormal SARC-F and/or low HGS	1.81 [1.03–3.19]	
Sarcopenia (Low HGS and low AC)	2.63 [1.41–4.91]	
Severe sarcopenia (Low HGS, low AC, and low PP)	3.37 [1.70–6.70]	

EWGSOP2, European Working Group on Sarcopenia in Older People 2019; HGS, hand-grip strength; AC, arm circumference; PP, physical performance; SARC-F, strength, assistance with walking, rising from a chair, climbing stairs, and falls. ^a^ Hazard ratios adjusted for age, in/out patient status, cancer site, metastatic status, cancer treatment, cognitive impairment, underweight, weight loss and number of prescription medications per day.

## Data Availability

Restrictions apply to the availability of these data. Data were obtained from the NutriAgeCancer Study Group and are available from the corresponding author with the permission of the NutriAgeCancer Study Group investigators.

## Supplementary Materials

The following supporting information can be downloaded at: https://www.mdpi.com/article/10.3390/nu15061508/s1, Figure S1: Study flow chart; Figure S2: Multivariate Cox analyses of the association between two composite variables for sarcopenia and 6-month mortality in patients with metastatic cancer who were able to perform the TUG test: A. A composite variable corresponding to the EWGSOP2 definition of sarcopenia; B. A composite variable that included the SARC-F in the definition; Supplementary Table S1. Multivariate Cox analyses of the association between low hand-grip strength and low arm circumference and 6-month mortality in in the subpopulations defined by the EWGSOP2 algorithm, overall and by metastatic status.

## References

[1] Cruz-Jentoft A.J., Bahat G., Bauer J., Boirie Y., Bruyere O., Cederholm T., Cooper C., Landi F., Rolland Y., Sayer A.A. (2019). Sarcopenia: Revised European consensus on definition and diagnosis. Age Ageing.

[2] Marhold M., Topakian T., Unseld M. (2021). Sarcopenia in cancer—A focus on elderly cancer patients. Memo Mag. Eur. Med. Oncol..

[3] Williams G.R., Rier H.N., McDonald A., Shachar S.S. (2019). Sarcopenia & aging in cancer. J. Geriatr. Oncol..

[4] Pamoukdjian F., Bouillet T., Levy V., Soussan M., Zelek L., Paillaud E. (2018). Prevalence and predictive value of pre-therapeutic sarcopenia in cancer patients: A systematic review. Clin. Nutr..

[5] Simmons L.O., Cagney D., Hassan F., Lim J.Y., O’Leary D.P., Liew A., Redmond H.P., Corrigan M., O’Sullivan M., Kelly L. (2019). Prevalence of sarcopenia and its impact on survival in breast cancer—A systematic review and meta-analysis. Mesentery Peritoneum.

[6] Catikkas N.M., Bahat Z., Oren M.M., Bahat G. (2022). Older cancer patients receiving radiotherapy: A systematic review for the role of sarcopenia in treatment outcomes. Aging Clin. Exp. Res..

[7] Xia L., Zhao R., Wan Q., Wu Y., Zhou Y., Wang Y., Cui Y., Shen X., Wu X. (2020). Sarcopenia and adverse health-related outcomes: An umbrella review of meta-analyses of observational studies. Cancer Med..

[8] Bozzetti F. (2017). Forcing the vicious circle: Sarcopenia increases toxicity, decreases response to chemotherapy and worsens with chemotherapy. Ann. Oncol..

[9] Shen Y., Hao Q., Zhou J., Dong B. (2017). The impact of frailty and sarcopenia on postoperative outcomes in older patients undergoing gastrectomy surgery: A systematic review and meta-analysis. BMC Geriatr..

[10] Chan M.Y., Chok K.S.H. (2019). Sarcopenia in pancreatic cancer—Effects on surgical outcomes and chemotherapy. World J. Gastrointest. Oncol..

[11] Kawaguchi Y., Hanaoka J., Ohshio Y., Okamoto K., Kaku R., Hayashi K., Shiratori T., Akazawa A. (2021). Does sarcopenia affect postoperative short- and long-term outcomes in patients with lung cancer?—A systematic review and meta-analysis. J. Thorac. Dis..

[12] Otten L., Stobäus N., Franz K., Genton L., Müller-Werdan U., Wirth R., Norman K. (2019). Impact of sarcopenia on 1-year mortality in older patients with cancer. Age Ageing.

[13] Malmstrom T.K., Morley J.E. (2013). SARC-F: A simple questionnaire to rapidly diagnose sarcopenia. J. Am. Med. Dir. Assoc..

[14] Hu F.J., Liu H., Liu X.L., Jia S.L., Hou L.S., Xia X., Dong B.R. (2021). Mid-Upper Arm Circumference as an Alternative Screening Instrument to Appendicular Skeletal Muscle Mass Index for Diagnosing Sarcopenia. Clin. Interv. Aging.

[15] Esteves C.L., Ohara D.G., Matos A.P., Ferreira V.T.K., Iosimuta N.C.R., Pegorari M.S. (2020). Anthropometric indicators as a discriminator of sarcopenia in community-dwelling older adults of the Amazon region: A cross-sectional study. BMC Geriatr..

[16] Xiang Q., Li Y., Xia X., Deng C., Wu X., Hou L., Yue J., Dong B. (2022). Associations of geriatric nutrition risk index and other nutritional risk-related indexes with sarcopenia presence and their value in sarcopenia diagnosis. BMC Geriatr..

[17] Williams G.R., Al-Obaidi M., Dai C., Bhatia S., Giri S. (2021). SARC-F for screening of sarcopenia among older adults with cancer. Cancer.

[18] Ezzatvar Y., Ramírez-Vélez R., Sáez de Asteasu M.L., Martínez-Velilla N., Zambom-Ferraresi F., Izquierdo M., García-Hermoso A. (2021). Physical Function and All-Cause Mortality in Older Adults Diagnosed with Cancer: A Systematic Review and Meta-Analysis. J. Gerontol. Biol. Sci. Med. Sci..

[19] Wijnhoven H.A., van Bokhorst-de van der Schueren M.A., Heymans M.W., de Vet H.C., Kruizenga H.M., Twisk J.W., Visser M. (2010). Low mid-upper arm circumference, calf circumference, and body mass index and mortality in older persons. J. Gerontol. A Biol. Sci. Med. Sci..

[20] Poisson J., Martinez-Tapia C., Heitz D., Geiss R., Albrand G., Falandry C., Gisselbrecht M., Couderc A.L., Boulahssass R., Liuu E. (2021). Prevalence and prognostic impact of cachexia among older patients with cancer: A nationwide cross-sectional survey (NutriAgeCancer). J. Cachexia Sarcopenia Muscle.

[21] Katz S., Ford A.B., Moskowitz R.W., Jackson B.A., Jaffe M.W. (1963). Studies of Illness in the Aged. The Index of Adl: A Standardized Measure of Biological and Psychosocial Function. JAMA.

[22] Podsiadlo D., Richardson S. (1991). The timed “Up & Go”: A test of basic functional mobility for frail elderly persons. J. Am. Geriatr. Soc..

[23] Folstein M.F., Folstein S.E., McHugh P.R. (1975). “Mini-mental state”. A practical method for grading the cognitive state of patients for the clinician. J. Psychiatr. Res..

[24] D’Ath P., Katona P., Mullan E., Evans S., Katona C. (1994). Screening, detection and management of depression in elderly primary care attenders. I: The acceptability and performance of the 15 item Geriatric Depression Scale (GDS15) and the development of short versions. Fam. Pract..

[25] Charlson M.E., Pompei P., Ales K.L., MacKenzie C.R. (1987). A new method of classifying prognostic comorbidity in longitudinal studies: Development and validation. J. Chronic Dis..

[26] Bellera C.A., Rainfray M., Mathoulin-Pélissier S., Mertens C., Delva F., Fonck M., Soubeyran P.L. (2012). Screening older cancer patients: First evaluation of the G-8 geriatric screening tool. Ann. Oncol..

[27] Malmstrom T.K., Miller D.K., Simonsick E.M., Ferrucci L., Morley J.E. (2016). SARC-F: A symptom score to predict persons with sarcopenia at risk for poor functional outcomes. J. Cachexia Sarcopenia Muscle.

[28] Roberts H.C., Denison H.J., Martin H.J., Patel H.P., Syddall H., Cooper C., Sayer A.A. (2011). A review of the measurement of grip strength in clinical and epidemiological studies: Towards a standardised approach. Age Ageing.

[29] Harrell F.E., Califf R.M., Pryor D.B., Lee K.L., Rosati R.A. (1982). Evaluating the yield of medical tests. JAMA.

[30] Gönen M., Heller G. (2005). Concordance probability and discriminatory power in proportional hazards regression. Biometrika.

[31] Yourman L.C., Lee S.J., Schonberg M.A., Widera E.W., Smith A.K. (2012). Prognostic indices for older adults: A systematic review. JAMA.

[32] Behne T.E.G., Dock-Nasimento D.B., Sierra J.C., Rodrigues H.H.N.P., Palauro M.L., Andreo F.O., Silva-The M.B., DE-Aguilar-Nascimento J.E. (2020). Association between preoperative potential sarcopenia and survival of cancer patients undergoing major surgical procedures. Rev. Col. Bras. Cir..

[33] Petermann-Rocha F., Balntzi V., Gray S.R., Lara J., Ho F.K., Pell J.P., Celis-Morales C. (2022). Global prevalence of sarcopenia and severe sarcopenia: A systematic review and meta-analysis. J. Cachexia Sarcopenia Muscle.

[34] Fernandes L.V., Paiva A.E.G., Silva A.C.B., de Castro I.C., Santiago A.F., de Oliveira E.P., Porto L.C.J. (2022). Prevalence of sarcopenia according to EWGSOP1 and EWGSOP2 in older adults and their associations with unfavorable health outcomes: A systematic review. Aging Clin. Exp. Res..

[35] Huang D.D., Cai H.Y., Wang W.B., Song H.N., Luo X., Dong W.X., Dong Q.T., Chen X.L., Yan J.Y. (2021). Measurement of muscle quantity/quality has additional predictive value for postoperative complications and long-term survival after gastrectomy for gastric cancer in patients with probable sarcopenia as defined by the new EWGSOP2 consensus: Analysis from a large-scale prospective study. Nutrition.

[36] Trussardi Fayh A.P., de Sousa I.M. (2021). Comparison of revised EWGSOP2 criteria of sarcopenia in patients with cancer using different parameters of muscle mass. PLoS ONE.

[37] Mori N., Maeda K., Fukami Y., Matsuyama R., Nonogaki T., Kato R., Ishida Y., Shimizu A., Ueshima J., Nagano A. (2022). High SARC-F score predicts poor survival of patients with cancer receiving palliative care. Support Care Cancer.

[38] Matsui M., Nishikawa H., Goto M., Asai A., Ushiro K., Ogura T., Takeuchi T., Nakamura S., Kakimoto K., Miyazaki T. (2021). Prognostic Impact of the SARC-F Score in Gastrointestinal Advanced Cancers. Cancers.

[39] Weng C.H., Tien C.P., Li C.I., L’Heureux A., Liu C.S., Lin C.H., Lin C.C., Lai S.W., Lai M.M., Lin W.Y. (2018). Mid-upper arm circumference, calf circumference and mortality in Chinese long-term care facility residents: A prospective cohort study. BMJ Open.

[40] Tsai A.C., Chang T.L. (2011). The effectiveness of BMI, calf circumference and mid-arm circumference in predicting subsequent mortality risk in elderly Taiwanese. Br. J. Nutr..

[41] Anjanappa M., Corden M., Green A., Roberts D., Hoskin P., McWilliam A., Choudhury A. (2020). Sarcopenia in cancer: Risking more than muscle loss. Tech. Innov. Patient Support Radiat. Oncol..

