# Supplementary material for: Prevalence of Four Sarcopenia Criteria in Older Patients with Cancer, and Their Predictive Value for 6-Month Mortality: The NutriAgeCancer National Prospective Cohort Study
Source: Nutrients. 2023 Mar 21;15(6):1508. doi: 10.3390/nu15061508 (PMC10051227; doi:10.3390/nu15061508)
Supplement: Supplementary file 1 [file nutrients-15-01508-s001.zip › nutrients-2268475-supplementary.pdf]

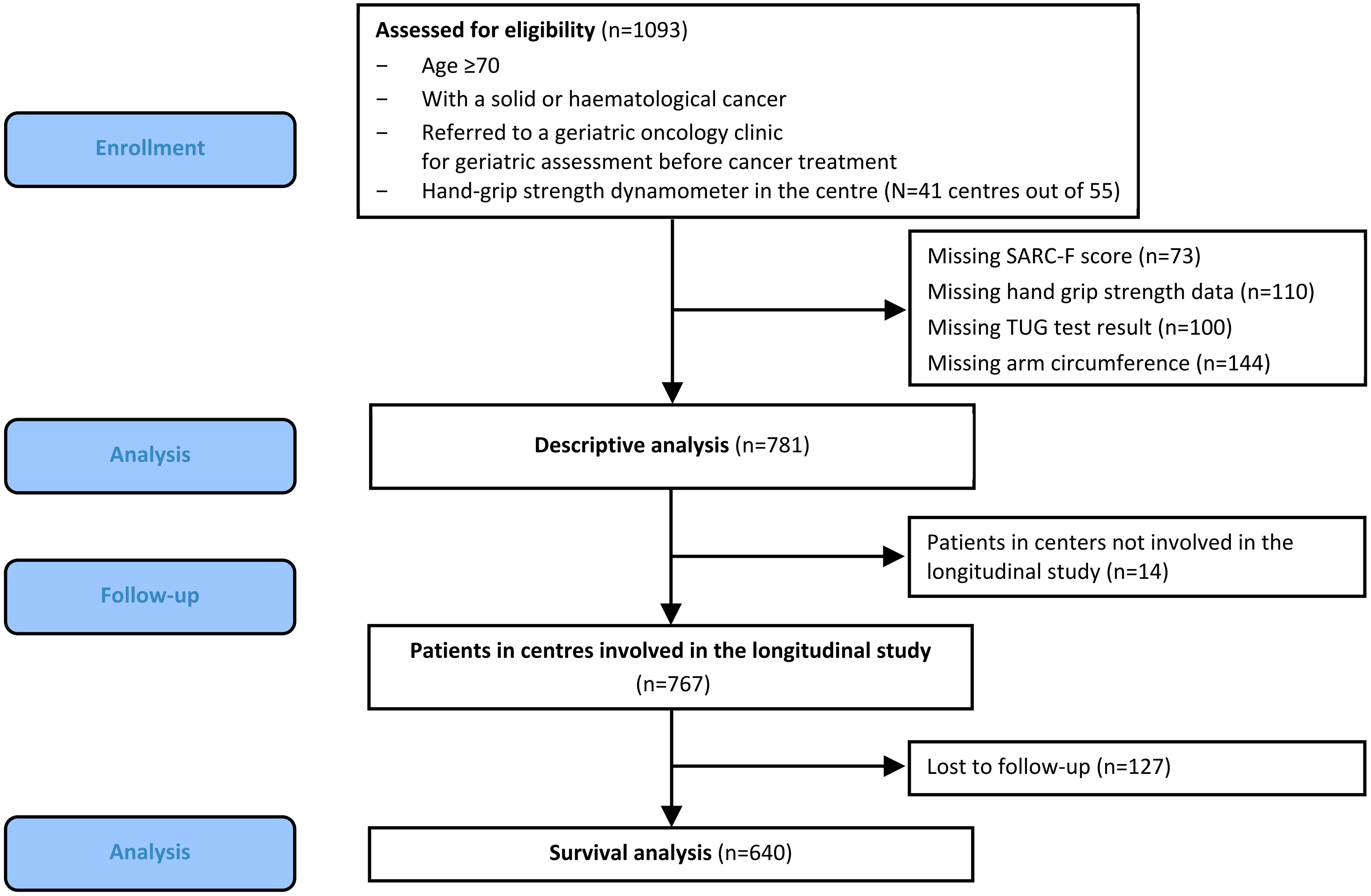

Supplementary Figure S1. Study flow chart

Supplementary Figure S2. Multivariate Cox analyses of the association between two composite variables for sarcopenia and 6-month mortality in patients with metastatic cancer who were able to perform the TUG test: **A.** A composite variable corresponding to the EWGSOP2 definition of sarcopenia; **B.** A composite variable that included the SARC-F in the definition.

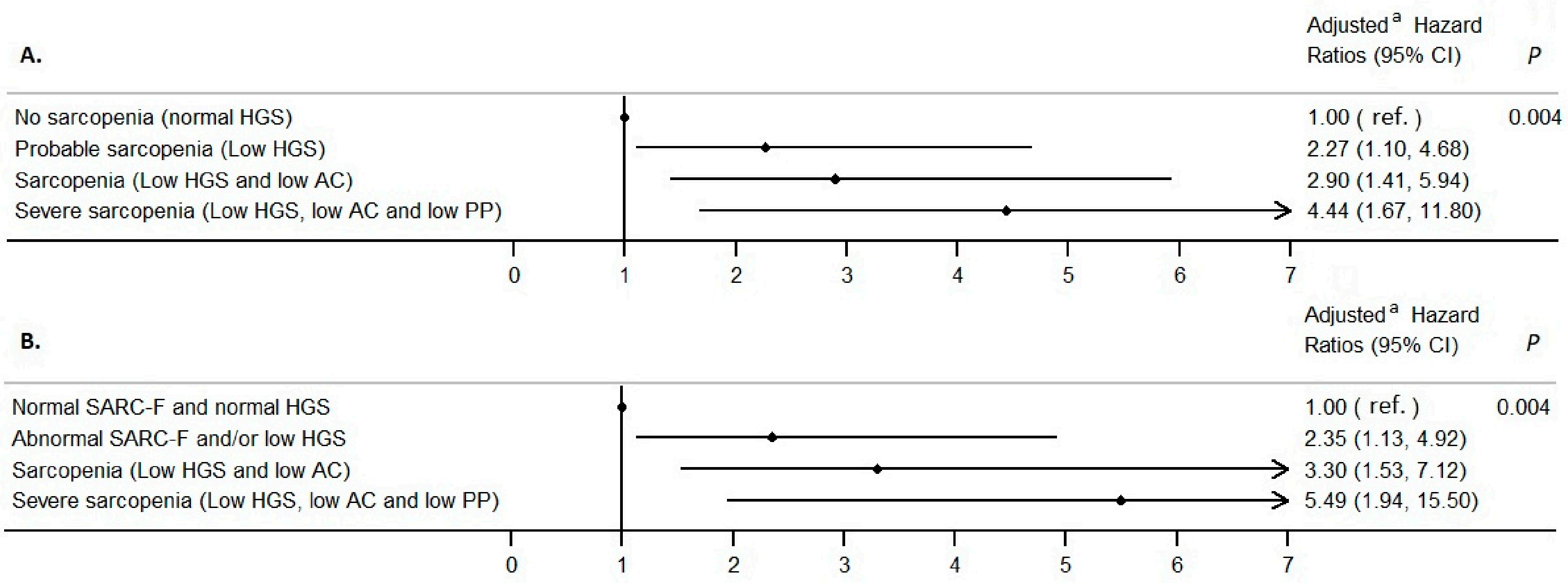

HGS, hand-grip strength; AC, arm circumference; PP, physical performance; SARC-F, strength, assistance with walking, rising from a chair, climbing stairs, and falls.

[a] Hazard Ratios adjusted for age, in/out patient status, cancer site, cancer treatment decision, cognitive impairment, underweight, weight loss and number of daily prescribed medications.

Supplementary Table S1. Multivariate Cox analyses of the association between low hand-grip strength and low arm circumference and 6-month mortality in in the subpopulations defined by the EWGSOP2 algorithm, overall and by metastatic status.

	**Overall population**			**Non-metastatic**			**Metastatic**		
	aHR	p-value	C-index	aHR	p-value	C-index	aHR	p-value	C-index
**Patients with an abnormal SARC-F** (n=223)									
Baseline model [a]	–		80.13	–		86.14	–		75.87
Low hand-grip strength	1.16 (0.65-2.08)	0.61	80.27	1.01 (0.37-2.74)	0.99	86.23	1.38 (0.61-3.12)	0.43	75.76
**Patients with an abnormal SARC-F and low HGS** (n=136)									
Baseline model	–		76.39	–		84.33	–		70.94
Low arm circumference	2.81 (1.28-6.19)	**0.010**	78.72	3.63 (0.84-15.6)	0.084	85.87	3.66 (1.09-12.3)	**0.036**	77.25

aHR, adjusted Hazard Ratios for variables in the baseline model; SARC-F, Strength, Assistance with walking, Rising from a chair, Climbing stairs, and Falls; HGS, hand-grip strength.

[a] Age (≥ 85 years), cancer site, metastatic status, cancer treatment decision (curative, palliative and supportive care), underweight (BMI <22 kg/m2), more than 5% of weight loss in the previous 6 months, number of daily prescribed medications, hospitalized vs. outpatient status and cognitive impairment (Impaired MMSE score (<24) or physician-diagnosed cognitive disorder).
